# Different distribution of *Helicobacter pylori EPIYA- cagA* motifs and dupA genes in the upper gastrointestinal diseases and correlation with clinical outcomes in iranian patients 

**Published:** 2015

**Authors:** Mohammad Hossein Haddadi, Abdollah Bazargani, Reza Khashei, Mohammad Reza Fattahi, Kamran Bagheri Lankarani, Maryam Moini, Seyed Mohammad Hossein Rokni Hosseini

**Affiliations:** 1 *Department of Bacteriology and Virology, Shiraz Medical School, Shiraz University of Medical Sciences, Shiraz, Iran*; 2 *Department of Internal Medicine, Gastroenterohepatology Research Center, Shiraz University of Medical Sciences, Shiraz, Iran*

**Keywords:** *Helicobacter pylori*, Gastric Cancer, *cagA* motifs, *dupA*, gastrointestinal disease

## Abstract

**Aim:**

Our aim was to determine the EPIYA-*cagA* Phosphorylation sites and *dupA* gene in *H. pylori* isolates among patients with upper gastrointestinal diseases.

**Background:**

Pathogenicity of the *cagA*-positive *Helicobacter pylori* is associated with EPIYA motifs and higher number of EPIYA-C segments is a risk factor of gastric cancer, while duodenal ulcer-promoting gene (*dupA*) is determined as a protective factor against gastric cancer.

**Patients and methods:**

A total of 280 non-repeated gastric biopsies obtained from patients undergoing endoscopy from January 2013 till July 2013. Samples were cultured on selective horse blood agar and incubated in microaerophilic atmosphere. The isolated organisms were identified as *H. pylori* by Gram staining and positive oxidase, catalase, and urease tests. Various motif types of *cagA *and the prevalence of *dupA* were determined by PCR method.

**Results:**

Out of 280 specimens, 128 (54.7%) isolated organisms were identified as *H. pylori*. Of 120 *H. pylori* isolates, 35.9% were *dupA* positive and 56.26% were *cagA* positive, while *cagA *with ABC and ABCC motifs were 55.5% and 44.5%, respectively. Fifty six percent of the isolates with the ABCC motif have had *dupA* genes. We also found a significant association between strains with genotypes of *dupA*-ABC and duodenal ulcer disease (*p *= 0.007).

**Conclusion:**

The results of this study showed that the prevalence of *cagA*-positive *H. pylori* in Shiraz was as high as in western countries and higher numbers of EPIYA-C segments were seen in gastric cancer patients. We may also use *dupA* as a prognostic and pathogenic marker for duodenal ulcer disease and *cagA* with the segment C for gastric cancer and gastric ulcer disease in this region.

## Introduction

*Helicobacter pylori* (*H. pylori*) is a spiral-shaped, Gram-negative bacillus which is known as the major cause of gastrointestinal diseases such as chronic gastric inflammation, ulcerative peptic diseases, gastric cancer (GC) and MALT-lymphoma (1). Chronicity of infection in more than 10% of the patients with *H. pylori* results in ulcer complication and in less than 1% may lead to GC. In addition, suffering from gastrointestinal diseases associated with *H. pylori* is influenced by various etiological factors such as bacterial strains, host genetics, and environmental conditions. Bacterial virulence factors have a crucial role in the development of various gastrointestinal diseases in different populations (2). Based on the presence of some virulence genes, *H. pylori* is divided into two types. Type I strains have a DNA region of several 40Kb genes called cag Pathogenicity Island (cagPAI) that encodes a type IV secretion system (TFSS) by which virulence proteins such as CagA (encoded by a gene in the pathogenicity island) delivers into the cytosol of gastric epithelial cells (3, 4). The *cagA* gene is considered as a marker to identify the cagPAI (5). The CagA protein induces morphological changes in host cells, which may be associated with the development of peptic ulcer and gastric carcinoma. As CagA enters into the host epithelial cell, phosphorylation of regulative proteins such as SH2 and SRC ensues. Therefore, it leads to increasing cell proliferation in the early stages of activating regulating cascades and finally leads to the cell apoptosis (6, 7).

 Protein CagA can affect cell activity in the way of its effects on the host signaling that are primarily attributed to the EPIYA motifs that are located in the C-terminus of the protein. The EPIYA motifs are comprised of 5 amino-acids Glu-Pro-Ile-Tyr-Ala, where the tyrosine residues serve as the site of phosphorylation. The numbers and types of the motifs repetition are different in the *cagA* genes and including EPIYA-A, -B, -C, and -D. EPIYA-A and -B motifs exists persistently in all positive *cagA*. However, the number and order of other two segments are different due to bacterial strains and pathogenicity power (6-8). The segments are ordered as EPIYA-A, -B, and -D in East Asian strains that are found in Central Asia, East Asian and North America, which have also been reported with a high incidence of gastric cancer cases, While, Western strains containing EPIYA-A, -B and -C motifs are observed in West and the Middle East countries, where the C motif may be duplicated multiple times and is different in various geographic regions (7, 8). Yamaoka, *et al*. found that as the number of repetitions of the segment C increases, bacterium will be more sensitive to pH of the environment and more potent in inducing the secretion of interleukin 8 (9)

There is an important gene region called plasticity region in some strains of *H. pylori* that is believed to contain about half of the virulence genes of this bacteria and encoding similar TFSS. The *dupA* gene (Duodenal ulcer promoting) is one of these genes, and is similar to the *virB* gene encoding TFSS. *dupA* is also an inducer for pro-inflammatory cytokine secretion of interleukin 8 (IL-8) and an activator of core transcription factor such as NFkB. Jung, *et al.* showed that about 80% of the strains of positive *dupA* have six other genes and *dupA* gene can be a marker to identify these genes that might play a role in producing secretory system similar to a secretory system of type IV(10-12). In some studies, *dupA* gene is introduced as a duodenal ulcer promoting factor. Additionally, positive *dupA* strains are more resistant to acidic conditions and have the ability to induce secretion of IL-8 more than other strains (11).

In this study, our aim was to find the distribution of various *cagA* motifs, prevalence of *dupA* in clinical* H. pylori* isolates, and to detect an association between these two virulence factors as well as various peptic diseases among the studied patients. 

## Patients and Methods

The ethics committee of Shiraz University of Medical Sciences approved this study and biopsy was taken from each patient voluntary and by filling testimonial. 

***Participants***

A total of 280 consecutive patients (164 male and 116 female) who underwent endoscopy due to upper gastrointestinal disorders at teaching hospitals (Nemmazi and Faqihi) in Shiraz, Iran from January to July 2013 were included in this study. Patients were grouped into four categories: 19 with gastric cancer (GC), 78 with gastric ulcer (GU), 80 with duodenal ulcer (DU) and 103 with non-ulcers disease (NUD) according to endoscopic and histological findings. None of the patients had received anti-inflammatory steroidal and antibiotic drugs during the three months prior to the sampling. (Table 1).

**Figure 1:**
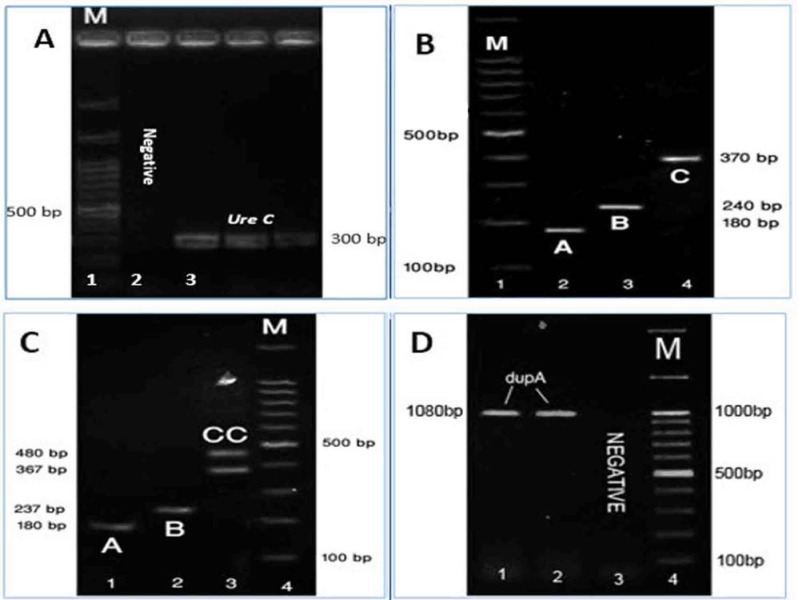
Characterized of *cagA* EPIYA motifs, *ureC* and *dupA* genes. A) *ureC* with 300 bp. B); Motif ABC, (1): motif A (180 bp), (2): 237 bp motif B (237 bp), (3): motif C (367 bp). C) Motif ABCC: motif A (180 bp), (2): 237 bp motif B (237 bp), (3): motif C (367 bp) and motif CC (480 bp). D) (1) and (2) are positive a sample of gene *dupA* with 1080 bp. (3): Patient with negative results. Lane (M) DNA ladder from 100-1500 bp.

**Table 1:** The distribution of *H. pylori* positive patients with various upper gastrointestinal diseases classified by gender and age.

Gender	Age (year)	Various gastrointestinal diseases				
		GC % (n)	GU % (n)	DU % (n)	NUD % (n)	Total % (n)
Male	15-25	0	11.1% (1)	22.2% (2)	66.7% (6)	11.82% (9/76)
	25-35	0	36.4% (4)	18.2% (2)	45.5% (5)	14.47% (11/76)
	35-45	0	27.3% (3)	36.4% (4)	36.4% (4)	14.47% (11/76)
	45-55	14.3% (2)	14.3% (2)	42.9% (6)	28.6% (4)	18.8% (14/76)
	55-65	25.0% (5)	25.0% (5)	15.0% (3)	35.0% (7)	26.3% (20/76)
	65-75	9.1% (1)	45.5% (5)	27.3% (3)	18.2% (2)	14.47% (11/76)
Female	15-25	0	0	50.0% (3)	50.0% (3)	11.5% (6/52)
	25-35	0	7.1% (1)	28.6% (4)	64.3% (9)	27% (14/52)
	35-45	0	45.5% (5)	18.2% (2)	36.4% (4)	21.2% (11/52)
	45-55	8.3% (1)	33.3% (4)	16.7% (2)	41.7% (5)	23% (12/52)
	55-65	50.0% (3)	16.7% (1)	33.3% (2)	0	11.5% (6/52)
	65-75	66.7% (2)	0	0	33.3% (1)	5.7% (3/52)
Total	-	2% (14)	25.8% (33)	24.2% (31)	40 (50)	1 (128/128)

**Table 2:** Primer sequences used in the study

Primer name	Sequence (5′-3′)	Amplification condition	Gene(s) ampliﬁed	Amplicon size (bp)
GlmM2-F	GGATAAGCTTTTAGGGGTGTTAGGGG	38 cycles: 45 s, 92°C; 1 min, 55°C and 45 s, 72°C	glmM	296
GlmM1-R	GCTTACTTTCTAACACTAACGCGC			
cag2 F	GGAACCCTAGTCGGTAATG	35 cycles: 45 s, 92°C; 1 min, 55°C and 45 s, 72°C	EPYIA 5´	
CagA-PIC R	GTCCTGCTTTCTTTTTATTAACTTKAGC		EPYIA-A	180
cagA-P2CGR	TTTAGCAACTTGAGCGTAAATGGG		EPYIA-B	240
cagA-P3E R	ATCAATTGTAGCGTAAATGGG		EPYIA-C	370
DupAF113 F	GACGATTGAGCGATGGGAATAT	35 cycles: 45 s, 92°C; 1 min, 57°C and 45 s, 72°C	*dupA*	1080

**Table 3:** Prevalence of *cagA* and various phosphorylation motif of EPYIA-*cagA* according to various gastrointestinal diseases

Various diseases	Various motifs of EPYIA*-cagA*							
	*cagA *Pos%(n)	*P* value *cagA*	ABC %(n)	*P* value ABC	ABCC%(n)	*P* value ABCC	*cagA *Neg %(n)	*P* value *cagA *Neg
GC	78.6 (11)	0.08	35.7 (5)	0.703	42.9 (6)	0.102	21.4 (3/14)	0.074
GU	57.6 )19)	0.859	24.2 (8)	0.313	33.3 (11)	0.199	42.4 (14/33)	0.859
DU	67.7 (21)	0.138	35.5 (11)	0.559	32.3 (10)	0.284	32.3 (10/31)	0.138
NUD	42 (21)	0	32.0 (16)	0.883	10.0 (5)	0.000	58.0 (29/50)	0.000
Total	56.2 (72)	0.03	31.3 (40)	0.784	25.0 (32)	0.01	43.8 (56/128)	0.03

***Culture***

Antral biopsy samples from each patient were placed in a fresh tioglycolate broth (Merck, Homburg, Germany) and transferred to the laboratory within less than 4 hours. After homogenizing, specimens were subsequently cultured on agar plates containing 7% horse blood (Faculty of Veterinary Medicine, Shiraz), fetal calf serum (FCS), vancomycin (10 µg/L), trimethoprim (5 µg/L), amphotericin B (5 µg/L) and antibiotics (sigma, USA). The inoculated plates were placed in the microaerophilic condition generated by Gas Pak jars (Merck, Homburg, Germany) and incubated for 5-7 days at 37° C. All of suspected colonies were confirmed as *H. pylori* by routine diagnostic tests including: Gram-staining, oxidase, catalase, and urease positive tests. DNA was isolated from confluent plate cultures expanded from a single colony using the QIAamp® tissue kit (Qiagen, Germany) according to the manufacturer's instructions. 

***PCR method***

Table 2 shows the Primers used in this study. For identification and veriﬁcation of *H. pylori* presence at the species level, the *ureC* gene was ampliﬁed using primers glmM, with a 296-bp size product for all samples (Fig 1.A) (12).

To assess the number and type of EPIYA motifs and to determine the presence of *dupA* gene, PCR was carried out using one set of specific primers for each gene segment (13, 14) (Table 2).

The expected lengths of PCR products were 180 bp for EPIYA-A, 240 bp for EPIYA -B, 370 bp and more for EPIYA -C, by using a single forward primer (cag2) for all motifs which shows that cag2 is specific for *cagA* gene (Fig 1.B, C). The *dupA* gene was ampliﬁed, using pairs of primers dupAF113-dupA AR1083 with a 1080-bp size product (Fig 1.D).

All PCR reactions were performed under a defined program using Ependrof Master Cycler 5530.(Eppendorf, Hamburg, Germany). 

Five microliters of the PCR product was electrophoresed on a 1.5% agarose gel, stained with ethidium bromide and analyzed by ultraviolet light trans-illuminator (EEC Company, England). For verifying the presence of each gene, twenty PCR products were sequenced and the nucleotide sequences of the amplified fragments were compared with nucleotide database of the National Center for Biotechnology Information database (http://www.ncbi.nlm.nih.gov/).

**Statistical analysis**

Statistical analysis was done by using SPSS version 21 software (SPSS, Chicago IL). Logistic-Regression, Chi-Square test and Fisher's exact test were used to analyze the data. Statistical values ​​p<0.05 was considered significant. 

## Results

Out of 280 gastric biopsies, 128 isolates (45.7%) were identified as *H. pylori* by routine biochemical tests and confirmed by the presence of *ureC *gene. Among them, 72 (43.9%) patients were male and 56 (48.27%) were female with a mean age of 26 years (range 15–75 years). *H. pylori* positive patients (128) were belonged to the following groups: 14/19 (73.6%) from GC, 33/78 (42.30%) from GU, 31/80 (38.7%) from DU and 50/103 (48.5%) from NUD. The presence of *dupA* gene and type of EPIYA motifs were also determined by PCR method. 

**The prevalence of** ***cagA*** **strains** **:** Table 3 shows the distribution of the *cagA* and EPIYA genotypes. Of 128 *H. pylori* -positive patients *cagA* positive isolates were observed in 56.26% (72/128) of them, of these, 57.9% (44/76) were male and 53.8% (28/52) were female. The presence of the *cagA* in the isolates from patients with GC, PUD (peptic ulcer disease including GU and DU) and NUD (Duodenitis and Gastritis) was 78%, 62.5% and 42%, respectively (Table 3). A significant association was seen between the presence of *cagA* gene and clinical outcome. (*p* = 0.034, OR = 1.52, 95% CI 1.07 to 2.17). In addition, our results indicate that patients with *cagA *strains are at risk for gastric cancer three times more than other patients. No significant relationship was observed between the prevalence of *cagA* and the disease due to small sample size of GC (*p* = 0.07 OR = 3.18, 95% CI 0.84 to 12.02).

***Distribution of the cagA motifs***

We also determined the distribution of the *cagA* motifs among 72 *cagA* positive *H. pylori* isolates**. **While *cagA *with EPIYA -ABC and -ABCC motifs were 55.5% (40/72) and 44.5% (32/72) respectively, no type of EPIYA -ABD, -ABCCC and -AB motifs was observed in our study. ABCC strains were the most prevalent, in patients with duodenal ulcer [35.5% (11/31)] and, patients with gastric cancer [42.9% (6/14)]. We found a significant association between the EPIYA -ABCC motif and PUD diseases. (*p *= 0.041 OR = 2.35, 95% CI 1.02 to 5.41) but, in this study no association between the EPIYA -ABC motif and PUD diseases was observed (Table 3).

**Table 4:** Distribution of positive *dupA* strains according to the disease being studied

*dupA *	Various diseases being studied							Total %(n)	
	GC%(n)	GU%(n)		DU%(n)		NUD%(n)			
*dupA *Pos	35.7 (5/14)		33.3 (11/33)		58 (18/31)		24 (12/50)		36 (46)	
*p* value	0.985		0.717		0.003		0.024		-	
*dupA *Neg	64.3 (9/14)		66.7 (22/33)		42 (13/31)		76 (38/50)		64 (82)	

**Table 5:** The distribution of *cagA*-*dupA* genotypes in the *H. pylori*-associated diseases

Distribution of genotypes	diseases				
	GC%(n)	GU%(n)	DU%(n)	NUD%(n)	Total%(n)
*dupA*+*cagA*+	28.6 (4)	18.2 (6)	38.7 (12)	10 (5)	21 (27)
*P* value	0.467	0.634	0.006	0.014	0.018
*dupA*+ ABC	0(0)	11.1 (1)	66.7 (6)	22.2 (2)	33 (9)
*P* value	0.276	0.297	0.002	0.283	0.021
*dupA*+ ABCC	22.2 (4)	27.8 (5)	33.3 (6)	16.7 (3)	66.7 (18)
*P* value	0.098	0.835	0.303	0.036	0.118
*dupA*+*cagA*-	5.3 (1)	26.3(5)	31.6 (6)	36.8 (7)	14.1 (19)
*P* value	0.390	0.954	0.417	0.830	0.756
*dupA- cagA+*	50 (7)	40 (13)	29 (9)	22 (11)	31.3 (40)
*P* value	0.131	0.241	0.760	0.071	0.147
*dupA-cagA-*	14.3 (2)	27.3 (9)	13 (4)	44 (22)	29 (37)
*P* value	0.201	0.810	0.024	0.003	0.012

***The prevalence of dupA strains***

 Out of 128 *H. pylori* *positive* samples, 46 (35.9%) were *dupA-*positive gene (Table 5). The prevalence of *dupA* gene in patients with DU (18/31, 58%) was higher than patients with GC (5/14, 35.7%), GU (11/33, 33.3%) and NUD (12/50, 24%). Statistical analysis showed a significant association between the presence of *dupA* and DU diseases (*p* = 0.03 OR = 3.14, 95% CI 1.47 to 7.8).

***Correlation between of the number of EPIYA-C motifs and the dupA genotype as well as clinical outcome***

 The distribution of the* H. pylori* isolates carrying both genes *cagA* and *dupA* were different in the studied peptic diseases. Twenty seven strains out of 128 strains were carrying both genes of *cagA* and *dupA* (21%). Out of all *cagA*-*dupA* positive strains, 38.7% (12/27) was from DU patients and 28.6% (4/27), 18.2% (6/27) and 10% (5/27) were from GC, GU and NUD patients, respectively. 

The presence of *dupA* genes among *cagA* strains with different EPIYA was studied and we found that *dupA*-ABC type were more prevalent among patients with DU 19% (6/31) compare to other patients. (Table 4)

 We also found a significant relationship between strains with genotypes of *dupA*-ABC and DU disease (*p *= 0.007 OR = 7.52, 95% CI 1.75 to 32.20) (Table 4). Fifty six percent of isolates with the ABCC motif have had *dupA* genes. A significant relationship was observed between the presence of *dupA *and ABCC (*p* = 0.02, OR = 2.61, 95% CI 1.15 to 5.94). Statistical analyses showed a significant relationship between genotype *dupA*-ABCC and GC (*p *= 0.03 OR= 3.88, 95% CI 1.02 to 14.7)(Table 4). 

## Discussion

The different size of *cagA* gene is correlated with the number of the repeated EPIYA-C motifs which is including phosphorylation sites and the increased incidence of upper gastrointestinal diseases such as gastric ulcer and gastric cancer (8, 15). The presence of *dupA* in plasticity region has been demonstrated as a high risk of development of DU worldwide (12, 16). The incidence differences in *cagA* gene can be explained by geographical differences. In Eastern countries such as China, prevalence of *cagA* has been reported as high as 89.3%, which may be in relation to the high prevalence of peptic diseases in Eastern populations. While in the countries of the Middle East such as Kuwait, Iraq, and Iran, the prevalence have been reported as 50%, 71% and 76%, respectively. In the countries such as Turkey and Spain, the presence of *cagA* gene in peptic diseases of PUD and GC has been reported as 71.6% and 37%, respectively (17-20). In the present survey, we studied the presence of *H. pylori* EPIYA-cagA motifs and *dupA* genes in the upper gastrointestinal diseases and determined their correlation with clinical outcomes. The prevalence of the *cagA* gene (56.26%) was close to our neighbor countries results such as Iraq and Saudi, with the prevalence of 48.57% and 51%, respectively (21, 22). In the previous study which was performed by Dabiri et al., the prevalence of *cagA* gene was reported 67% in Iran (23) Furthermore, the prevalence of strains with repeated C segment (44.5%) was much higher than the similar study in Iran and Iraq (16.75% and 3.3%), but was closer to 34% in Colombia (22, 24, 15).

 However, in our study we did not find any EPIYA-AB, -ABCCC and -ABD motifs, that was in contradiction with Kargar, *et al*. report (25). This may be due to a small number of patients with severe diseases in the current study. This study and other studies by Shokrzadeh, *et al *and Vaziri, F, *et al*. (24, 26) in Iran suggest that high prevalence of strains with repeated C segments, increase the risk of GC development. Similar finding was reported by Batista, *et al*.(14, 8). Results of our study was consistent with those of Hussein, et al. in which increase of segment C in strains with ulcer diseases is more than strains with non-ulcer diseases (26- 29).

Previous studies by Vaziri et al. showed that a minority of *H. pylori* strains (4%) may show AB/C motif due to recombination events. The pathogenesis effect of AB/C motif is intermediate of A and B motifs. However, all *cagA* positive strains show the basic AB motif type. Moreover, another motif called Amerindian, that is the result of recombination in short sequences (10-12 bps) in AT rich region, may be produced. This motif has an intermediate effect between C and D motifs. This recombination does not make a significant change in the length of the segments, which were used in our method. The method proposed by Argent et al. was used in our study to evaluate each motif separately. However, in the previous studies, the 3’ terminal region was amplified and therefore for motif typing, sequencing was necessary which is costly and time consuming (13, 26, 28).

To the best of our knowledge, since 2008 until now, only one study has evaluated the presence of *dupA* and *cagA*-motifs simultaneously. While several studies have determined only the prevalence of *dupA* or *cagA* genes (29), there are two main reasons for studying the presence of *dupA* and type distribution of *cagA* motifs. First, *dupA* is one of the genes in plasticity region that includes half of the virulence genes in strains 26695 and J99, and *cagA* strains (as a marker cagPAI) are more virulent than other strains. Second, *dupA* is determined as a protective factor against GC and more resistant in the acidic pH while EPIYA motifs with repeated of C segment is correlated with GC and highly sensitive against acidic pH (16, 11, 8).

In the present study, the presence of *dupA* was lower than previous studies reported from Iran that might be due to the small sample size in our study. However, the prevalence of *dupA* in China was 35.5% that is similar to our study (16, 30).

Our statistical analyses showed that the presence of *dupA* increases the risk for development of duodenal ulcer compared to other diseases. Previous study in Iran also reported the similar finding (29, 16). In our study, DU disease was associated with a high frequency of strains possessing *dupA* genes with EPIYA-ABC, however, this result was not found in other studies (28). We found that strains with *dupA* were associated with DU and carrying EPIYA-ABC and *dupA* increase the risk of DU, while the strains with -ABCC and *dupA* were associated with GC. These results also confirmed the previous studies and showed that strains with -ABC and *dupA* were more resistant to acidic conditions of the antrum region than strains with –ABCC and these samples have a greater ability to create an ulcer in the duodenum region. Results of this study also showed, that creating ulcer in the duodenum region is associated with the presence of *dupA* and these strains increase the risk of duodenal ulcer by having –ABC motif in comparison to having more numbers of fragment C, possibly with different resistance to acidic conditions. Whereas, in GC and GU, more numbers of fragment C increase the risk of disease while the secretion of acid in this region is less than the duodenum region. It is noteworthy that the presence of *dupA* was not associated with the diseases of the corpus region. 

According to the results of this study and other similar studies, it can be concluded that in the environment with low pH, the presence of *dupA* likely increases the survival of *H. pylori* by unknown mechanism and producing a lasting secretory system to secret *cagA* protein may be effective in the process of creating ulcer disease in this region. This hypothesis is based on the findings that strains with –ABCC motif are more sensitive to low pH and have a lower survival power in the region. Therefore, *dupA* resistance to acidic pH has no effect on the survival of these strains and more resistant strains containing –ABC motif have the selection power in this area. 

The results of this study indicate that the risk of cancer with strains containing segment C is more than other strains in our region and we can use *dupA* as a prognostic and pathogenic marker for duodenal ulcer. We may also use EPIYA with the fragment C as a risk factor for gastric cancer and duodenal ulcer in our area.
